# Description of Acute and Chronic Cases of Poisoning by *Oxalis pes-caprae*

**DOI:** 10.3390/ani15111668

**Published:** 2025-06-05

**Authors:** Luigia Pinna, Daniela Mandas, Davide Pintus, Bruna Zulato, Marina Frongia, Maria Maurichi, Annamaria Coccollone

**Affiliations:** 1 S.C. Complex Territorial Diagnostic Structure of Cagliari, Istituto Zooprofilattico Sperimentale of Sardinia, Via dell’Acquedotto Romano, 09030 Elmas-Cagliari, Italy; luigia.pinna@izs-sardegna.it (L.P.); bruna.zulato@izs-sardegna.it (B.Z.); marina.frongia@izs-sardegna.it (M.F.); maria.maurichi@izs-sardegna.it (M.M.); annamaria.coccollone@izs-sardegna.it (A.C.); 2 S.S. Anatomo-Histopathology and Animal Genetics, Sassari Section, Istituto Zooprofilattico Sperimentale of Sardinia, Via Vienna 2, 07100 Sassari, Italy; davide.pintus@izs-sardegna.it

**Keywords:** *Oxalis pes-caprae*, sheep grazing, poisoning

## Abstract

*Oxalis pes-caprae* is one of the most widespread invasive plants in the Mediterranean areas, especially in central and southern Italy, Sicily, and Sardinia. It is an herbaceous species of South African origin, very common in uncultivated areas, in meadows, and at the edges of roads but also in vegetable gardens and fields, where it can become a weed. Its negative impact on the economic sector is significant due to the presence of large quantities of oxalates, which are toxic and dangerous for grazing livestock; the ingestion of high amounts of oxalates causes the accumulation of oxalate crystals, which can lead to kidney failure and anuria. This work represents a descriptive, field-based case report of epidemiological relevance, describing two cases of acute and chronic poisoning by *Oxalis pes-caprae* found in two different sheep farms in southern Sardinia. In February 2024, the sudden death of about 40 animals was reported in a sheep farm. On another farm, seven animals died (between March and July 2024), while others showed poisoning symptoms such as weight loss, submandibular edema, and a barrel-shaped abdomen. This manuscript aims to highlight the damage caused by poor attention in pasture management and the importance of seasonal risk management; it is necessary to pay attention to the herbaceous species present in pastures, especially in our region, where climatic temperatures no longer respect seasonality and herbaceous species that normally grow in spring are easily found also in the winter months.

## Simple Summary

African wood-sorrel (*Oxalis pes-caprae*, Oxalidaceae family) was first introduced into the island regions of Italy and other Mediterranean regions toward the end of the 18th century, and it is now present throughout the central–southern part of our country. *Oxalis pes-caprae* blooms in winter and continues to do so until spring. This plant contains high concentrations of oxalic acid, mainly in the flower scapes, which can cause oxalic acid poisoning in grazing animals and humans if ingested in large quantities. This work describes two cases of *Oxalis pes-caprae* poisoning found in two different sheep farms in southern Sardinia. We emphasize the importance of maintaining controlled feeding in pastures.

## 1. Introduction

**Figure 1:**
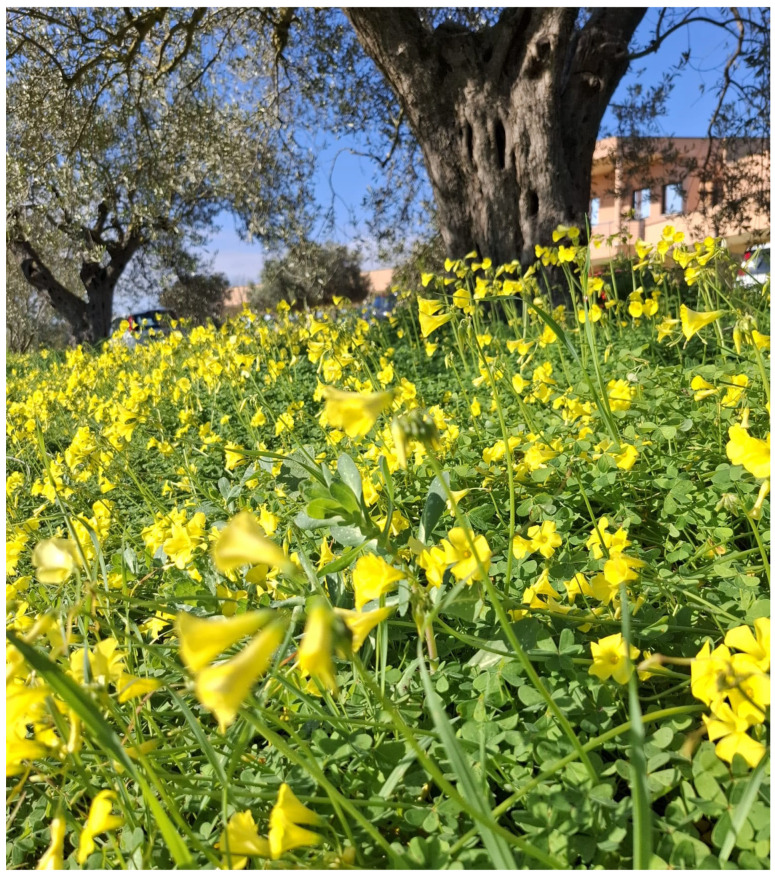
Growth of *Oxalis pes-caprae* on roadsides.

Among species of weeds found in the Mediterranean basin, *Oxalis pes-caprae* is one of the most widespread, especially along central and southern Italy, Sicily, and Sardinia. It is a weedy botanical species belonging to the Asteraceae family and is a plant of South African and Namibian origin, also known as African wood-sorrel [1,2]. It was first introduced to Europe in 1757, mainly for ornamental purposes, and spread throughout the Mediterranean basin, from Sicily in 1796 to Sardinia in 1859 and Crete in 1883. It is a highly competitive botanical species with a strong impact on native biodiversity; it is widely distributed in moist and shaded soils, in woods, along the edges and banks of watercourses, in the plains, and in the submontane belt, within thermo- and meso-Mediterranean bioclimatic zones. It is also included in the list of the 100 most invasive species of our continent (Figure 1) [3,4].

*Oxalis pes-caprae* produces numerous bulbils in spring as part of its vegetative reproduction. These bulbils germinate in autumn and begin to bloom from winter until the following spring. Its abundant spring flowering has a strong positive impact on the esthetic and landscape aspects of the invaded areas. However, its effects on the economic sector are particularly concerning. The petioles and leaves contain large quantities of oxalates, which are toxic and dangerous for livestock; several reports in the literature describe toxic effects on cattle and sheep in areas where pastoral production is widespread. In fact, poisoning due to the ingestion of high quantities of these plants by herbivores is quite widespread [5,6,7,8,9,10].

The major oxalate-producing shrubs and herbaceous plants worldwide belong to the genus *Rumex* or genera of the *Chenopodiaceae* and *Oxalidaceae* families. Other oxalate-containing plants include several species of the *Agave, Beta*, *Bassia*, *Halogeton*, *Rhuem*, *Rumex*, *Sarcobatus*, *Setaria*, and *Amaranthus* genera, which are found throughout the world [11,12,13].

Oxalic acid is an alpha, omega-dicarboxylic acid readily soluble in water, alcohol, and ether, which crystallizes with two water molecules in monoclinic, odorless, and acid–metallic-tasting needles. Toxic doses of oxalates from plants depend on several factors; according to some authors, a lethal dose for sheep with adequate nutrition is 5–6 g/kg of body weight. This value decreases to 1 g/kg in animals with poor nutrition and limited water intake. The degree of intoxication also depends on the speed of ingestion, so the quantities indicated can be significantly reduced. In this context, an important role is played by the microbial flora of the rumen, which can increase the organism’s tolerance of oxalates by up to 30% following repeated contact with these plants. In fact, ingested oxalates can be completely degraded in the rumen with the formation of carbonate and bicarbonate radicals, which cause an increase in environmental pH, or they can precipitate in the form of calcium salts and eliminated with feces [7,11]. Oxalate toxicity depends on the affinity of the oxalate ions for the calcium ions, which leads to the formation of insoluble calcium oxalate, making calcium unavailable for absorption. Acute, subacute, or chronic poisoning and its severity are conditioned by the quantity of soluble oxalates absorbed into the bloodstream and consequently by the botanical species consumed [7,14].

The symptoms reported in the literature describe severe hypocalcemia in acute poisonings, while in subacute or chronic cases, moderate hypocalcemia is mainly observed, followed by skeletal alterations, particularly during growth phases. Crystalline infiltrations of oxalates have also been observed in the walls of blood vessels, where they cause necrotic and hemorrhagic episodes. In the kidneys, insoluble oxalate precipitation occurs within the tubules, where the accumulation of oxalate crystals can cause kidney failure and anuria [7,15].

Alterations in clinical–chemical parameters have also been detected: hyperphosphatemia, increased blood levels of sodium and potassium, and increased serum AST, ALT, LD, and urea nitrogen.

In acute cases, symptoms appear 2 to 6 h after the ingestion of plants containing oxalates (*Oxalis* spp.). If the ingestion of plants containing acid oxalates has occurred, death may occur within 9 to 11 h of the onset of symptoms. In acute intoxication cases, the predominant clinical picture is attributable to hypocalcemia, which manifests primarily through neurological symptoms [7].

The lesions found during necropsy primarily affect the digestive system, characterized by irritation and inflammation of the mucous membranes, edema formation, and hemorrhages. Furthermore, the kidneys appear pale, edematous, enlarged, and easily decapsulated. Ascitic effusions and hydrothorax are frequently observed, often accompanied by petechial hemorrhages on the serous membranes. The lymph nodes appear enlarged and edematous. Generally, these lesions are closely related to the number of toxic agents ingested [7,13,16].

Histological examination reveals needle-like calcium oxalate crystals within the walls of blood vessels and abdominal viscera. The accumulation of crystals is also observed in the renal tubules, often associated with their dilation and rupture. In the cortico-medullary area, accumulations of oxalate crystals can be appreciated, and in the most severe cases, they are macroscopically visible [7,11,13,16].

In Italy, documentation of cases of poisoning by *Oxalis pes-caprae* is scarce; the few available works are communications to national conferences, such as the works of Stelletta C. et al. and Puleio R. et al. presented at the SIPAOC conference in 2012 [17,18] and of Coccollone A. et al. presented at the SIPAOC conference in 2024 [19].

This manuscript describes the lesions found in animals that died following the ingestion of *Oxalis pes-caprae*. The evaluation of the breeding and grazing context and, subsequently, of the anatomo-pathological, histological picture and the results of the analyses carried out on biological fluids led to the diagnosis of oxalate poisoning.

The description concerns two mortality episodes that occurred in two different sheep farms located in the province of Cagliari, in South Sardinia (Italy), respectively, in February 2024 and March–July 2024; it has been hypothesized that it could be acute intoxication in the first case, in which the sudden death of several animals was recorded, and chronic intoxication in the second case, where the death of a few animals occurred over a longer period of time and the presence of symptomatic animals was observed in the farm. Diagnostic analyses performed on dead animals confirmed the accumulation of oxalate crystals and several related pathological alterations.

## 2. Case Report

Two sheep farms, located in different municipalities in the province of Cagliari, South Sardinia (Italy), were affected by cases of mortality. In February 2024, an episode of sudden mortality affecting approximately 40 sheep occurred in one of the two farms; 2 dead sheep were delivered to IZS Sardinia for diagnostic investigations. The sheep were in good nutritional condition: one was an adult, and the other was young and pregnant. This farm consisted of about 350 sheep. In the morning and evening, they were usually fed on pasture, supplemented with fodder, while at night, they were provided with straw. During the period when the sudden mortality episode occurred, the pastures were particularly rich in *Oxalis pes-caprae*, probably due to the favorable spring temperatures.

In the other episode analyzed in this report, the death of seven sheep was recorded between March and July 2024; two dead sheep were delivered to IZS Sardinia for diagnostic investigations. Both sheep were adults, and they had poor nutritional status. The other animals on the farm showed symptoms such as weight loss and depression, which lasted for several months. This farm consisted of approximately 200 sheep; the main source of food was grazing within citrus groves, where *Oxalis pes-caprae* grows abundantly. Additionally, hay, fodder, and pelleted alfalfa were administered as dietary supplements.

In both cases, farmers reported that grazing animals ingested large amounts of *Oxalis pes-caprae*, the prevalent herbaceous plant in those pastures.

## 3. Materials and Methods

A total of four animals were examined: two sheep from the February 2024 episode and another two sheep from the March and July 2024 episode. All the sheep were delivered to the Istituto Zooprofilattico Sperimentale of Sardinia (IZS Sardinia), Cagliari Section, for laboratory diagnostic tests and necropsy.

Several organs (see Table 1) were sampled for bacteriological and histological examinations.

Bacteriological cultural examinations were performed via the agar plate culture method with an incubation temperature of 37 °C, using an agar blood medium, a MacConkey agar medium, and nutrient broth.

For histological exams, collected organs were fixed in 10% formalin and embedded in paraffin. According to routine laboratory protocols, 4 μm thick sections were stained using the ST Infinity Haematoxylin & Eosin Staining System (Leica Biosystems, Richmond, IL, USA) and then observed using a light microscope (from 50× to 400× magnification).

Moreover, biochemical parameters of urea and calcium were measured in the collected urine samples (see Table 1), following the protocol described by the unpublished internal method MI 0DC/01 “Biochemical parameters on biological fluids of different animal species: determination by automated spectrophotometer”, using an automated spectrophotometer (Dimension^®^ EXL™ 200 Siemens, Munich, Germany).

Finally, biochemical parameters were determined for a blood sample (albumin, ALP, total bilirubin, calcium, creatinine, phosphorus, total proteins, GGT, AST, ALT, urea, cholesterol, CPK, glucose, and triglycerides) of a dead sheep from the second episode (see Table 1); an automatic spectrophotometer (Dimension^®^ EXL™ 200 Siemens, Munich, Germany) was also used in this type of analysis, following the protocol described by the internal method MI 0DC/01 “Biochemical parameters on biological fluids of different animal species: determination by automated spectrophotometer”.

Furthermore, in the second affected farm, blood samples were taken from five live animals to determine the leukocyte formula (%neut, %lymph, %mono, %eos, %baso, #neut, #limph, #mono, #eos, #baso, #luc, %luc, WBC, RBC, HGB, HCT, MCV, MCH, MCHC, and PLT) using automatic blood counting (Abbott Cell-Dyn 3700 Hematology Analyzer, Bimedis, Kissimmee, FL, USA) and biochemical parameters (albumin, calcium, creatinine, phosphorus, total proteins, and urea) using an automated spectrophotometer (Dimension^®^ EXL™ 200 Siemens, Munich, Germany), as indicated in Table 1. These five sheep were chosen based only on the symptoms observed in the herd (onset of weight loss and depression).

## 4. Results

**Figure 2:**
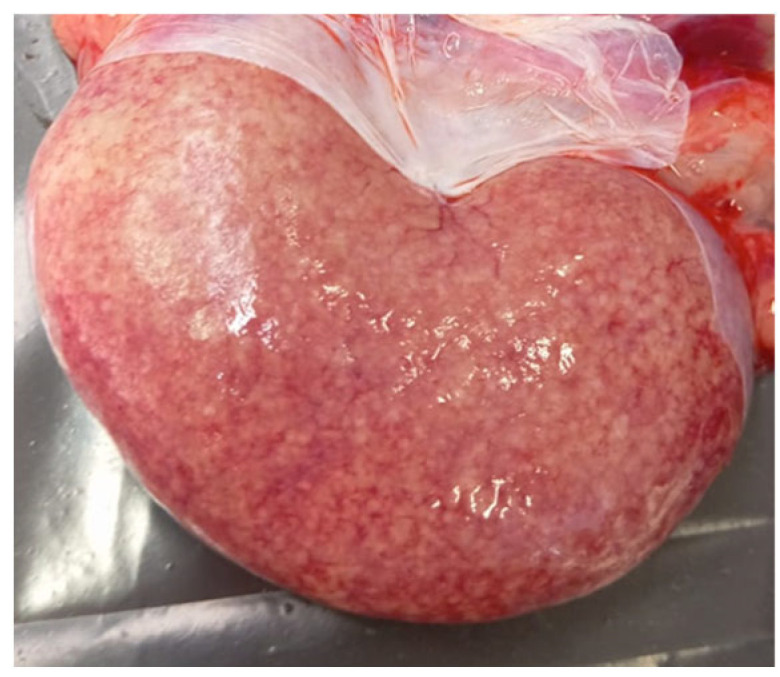
Pale kidney with hemorrhagic suffusion on the surface.

The pathological examinations performed on two dead animals from the first episode showed rumen tympanism and mild ruminitis associated with severe hemorrhagic abomasitis and enteritis. Moreover, the mesenteric lymph nodes appeared brownish and enlarged. Furthermore, the liver was hard in consistency with suffusions on the surface, and the kidneys appeared pale and soft, containing multiple hemorrhagic suffusions on the cortex (Figure 2).

Histologically, multifocal tubulonephrosis/tubulonecrosis and the accumulation of oxalate crystals were observed in the lumen of some tubules. These changes were associated with glomerular congestion and the presence of some atrophic glomeruli. Based on these histopathologic aspects, a toxic state possibly caused by *Oxalis* spp. was suspected. In the liver, hepatocytes exhibited swelling and degeneration characterized by a loss of cellular boundaries. Moreover, mild lymphocytic infiltrates were also observed. Culture tests performed on the brain and mesenteric lymph node did not reveal any microbiological abnormalities, while biochemical parameter tests performed on urine samples revealed very high urea values and extremely low calcium values, as reported in Table 2.

**Figure 3:**
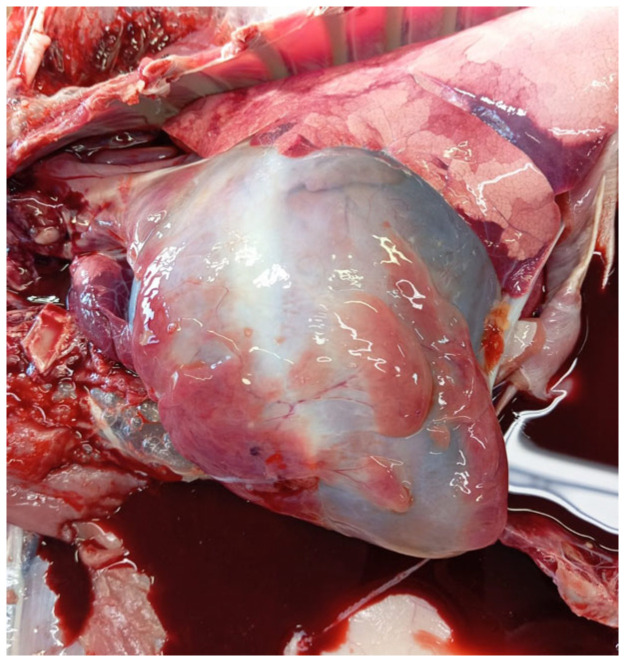
Gelatinous atrophy of the adipose tissue of the heart.

**Figure 4:**
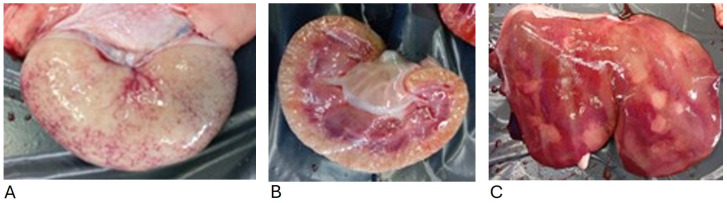
Pale kidneys, with irregular surfaces and increased consistency (**A**,**B**); hepatomegaly with multifocal grayish areas of irregular shape (**C**).

In March and July 2024, on the second farm, two sheep were submitted for necroscopy. In the first case, poor nutritional status and gelatinous atrophy of the adipose tissue at the base of the heart, indicative of a cachectic state, were revealed, as shown in Figure 3. Additionally, anemic mucous membranes and edema in the neck, submandibular region, and eyelids were observed. The abdomen appeared dilated, and abundant effusion was observed in the serous cavities (pleural, pericardial, and peritoneal). The kidneys were pale and atrophic, with an irregular surface and a gritty cut surface (Figure 4A,B); hepatomegaly with multifocal irregularly shaped grayish areas was also observed (Figure 4C).

The second sheep submitted for necropsy showed catarrhal abomasitis. The mesenteric lymph nodes were enlarged, and hemorrhagic petechiae were observed in the mesentery. The kidneys were small and pale with irregular surfaces; the cortex and medulla showed gritty cut surfaces. The liver appeared enlarged, with rounded gray areas extending into the parenchyma. There was serous fluid in the thoracic cavity, hydropericardium, and cardiac petechial hemorrhage.

**Figure 5:**
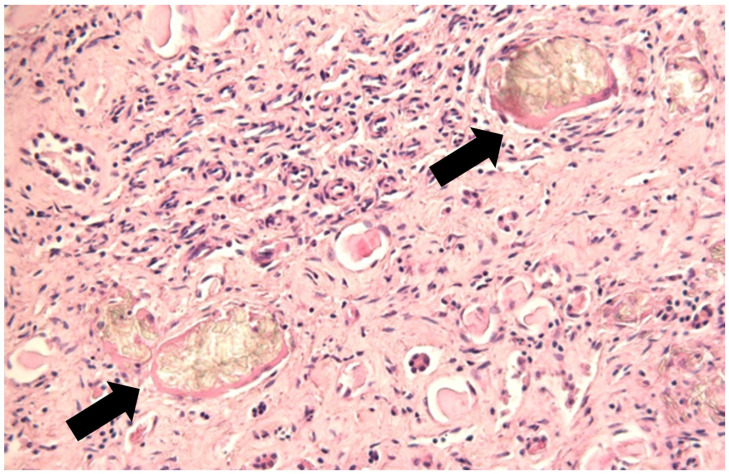
Kidney: accumulation of calcium oxalate crystals in the tubules (black arrow); severe interstitial fibrosis with tubular atrophy (H&E).

Histologically, similar findings were observed in the kidneys and liver of both animals undergoing necropsy. Chronic interstitial glomerulonephritis characterized by oxalate crystals in numerous tubules, the thickening of the wall of Bowman’s capsule, severe interstitial fibrosis with tubular atrophy, and multifocal lymphocytic infiltrates (Figure 5) consistent with intoxication by *Oxalis* spp. were determined. Histological liver sections showed necrotic–hemorrhagic areas with congestion of the portal vein. Bacteria culture tests performed on the brain, kidneys, intestine, lung, liver, and mesenteric lymph node did not reveal any microbiological abnormalities, while the evaluation of biochemical parameters in the blood showed some altered values, including urea, as shown in Table 3.

Finally, culture tests performed on the brain, liver, kidney, and mesenteric lymph node did not reveal any microbiological abnormalities.

Furthermore, the leukocyte formula and biochemical parameters were determined for live animals in this farm; they showed a platelet (PLT) level lower than the reference value in four of the five tested animals, and only one of the five tested animals showed slight alterations in both urea and calcium, as shown in Table 4.

## 5. Discussion

The clinical cases described in this work, which occurred in February, March, and July, are attributable to episodes of poisoning by *Oxalis pes-caprae*. The genus *Oxalis* includes plants with a high oxalate content that are widespread globally. The toxicity of soluble oxalates is species-dependent; monogastric animals respond differently than ruminants. Ruminants are generally less susceptible to chronic poisoning, but they are poisoned when they ingest plants containing relatively high oxalate concentrations without sufficient time for physiological adaptation [13]. In the cases described, both types of poisoning, acute and chronic, could be observed.

According to the literature, acute cases of *Oxalis pes-caprae* poisoning cause severe hypocalcemia; this factor also emerged from the analyses performed on the urine of the sheep that died in the first episode, as previously described (Table 2). As can be seen in Table 4, the analyses carried out on blood samples from five live animals highlighted a sample with a slight alteration in calcium (7.5 mg/dL–REF 8–12 mg/dL) and urea (111 mg/dL–REF 25–60 mg/dL), probably attributable to the onset of *Oxalis pes-caprae* intoxication. Also, lowering PLT levels can be observed in four of the five blood samples (Table 4). Furthermore, another crucial sign of this type of poisoning is the accumulation of insoluble oxalate precipitates in the renal tubules; abundant formation of oxalate crystals can cause renal blockage and consequent anuria [7,11].

In the first cases analyzed in this work, tubulonephrosis/tubulonecrosis accumulation of oxalate crystals in the lumen of some renal tubules and glomerular congestion were histologically observed (as described in paragraph 4). Externally, the kidneys appeared pale and slightly diminished in consistency, with hemorrhagic suffusions on the cortex, a characteristic also reported by Aslani [20]. These renal alterations, particularly the accumulation of oxalate crystals, correspond to the lesions described in the literature by several authors, associated with poisoning by oxalic acid contained in numerous plants [11,15,20]. As reported in the literature, if the concentration of oxalate ions becomes very high in the blood filtered by the kidney, it can combine with Ca^2+^ or Mg^2+^ to form insoluble oxalate crystals, which can block the flow of urine and cause kidney failure [21].

The second case presented a lesion similar to the one described previously and, as also described by the authors Naudè and Naidoo (2007) and Panciera RJ (1990), the thickening of the wall of Bowman’s capsule, severe interstitial fibrosis with tubular atrophy, and, sometimes, multifocal lymphocytic infiltrates (Figure 5) [11,15]. Furthermore, the cortex and medulla displayed increased firmness and a gritty texture upon sectioning. The lesion was attributable to chronic sclerosing interstitial glomerulonephritis, compatible with intoxication by *Oxalis* spp.

The clinical and pathological findings are consistent with descriptions in the literature of oxalate poisoning in sheep, in which hypocalcemia, azotemia, and nephropathy associated with the precipitation of calcium oxalate crystals in the renal tubules are characteristic findings [15,22]. Furthermore, other values have undergone strong alterations, such as GGT, AST, and ALT, which indicate liver malfunction, and the CPK value, which indicates muscle damage, as reported in Table 3.

Acute oxalate poisoning depends on several factors, including the total amount of oxalates consumed, adaptation to the oxalate-containing diet, and the amount and quality of other foods consumed at the same time [13,16,23]. However, ruminants tolerate a higher quantity of oxalates in their diet than other animals because the rumen microflora easily metabolizes some of them [20,21,24]. Furthermore, over time, the microorganisms of the rumen microflora involved in the degradation of oxalates increase with gradual exposure to increasingly higher concentrations, allowing ruminants to consume significantly larger quantities of oxalic acid-containing plants [14,20,23]. However, it is necessary to pay attention to animal nutrition, because plants containing oxalates are widespread and oxalates can remain if plants are preserved in hay or other prepared feeds. These high-oxalate-containing plants occur worldwide and include various species of the *Agave*, *Beta*, *Bassia*, *Chenopodium*, *Halogeton*, *Oxalis*, *Rhuem*, *Rumex*, *Sarcobatus*, *Setaria*, and *Amaranthus* genera [11,13].

Furthermore, a similar type of poisoning can be observed in animals following the ingestion of ethylene glycol; some authors have analyzed the clinical and morphopathological aspects of ethylene glycol poisoning, identifying the contamination of pastures with fluids from tractor tires as a risk for the ruminant category. Ethylene glycol (EG) is a toxic substance commonly found in products such as antifreeze, brake fluid, and other industrial products. Due to its biotransformation in the liver in the presence of alcohol dehydrogenase, toxic metabolites are formed which cause metabolic disorders and kidney damage with the accumulation of oxalate crystals in the tubules, as in oxalate poisoning due to plant ingestion [13,25,26]. This is not true of the case described in this manuscript, because the pastures we analyzed are not subjected to tractor treatments.

Finally, the diagnoses of the cases described in the manuscript are based exclusively on the anamnesis provided by the breeders and on the results of clinical and pathological investigations. Although the reported clinical and pathological findings are consistent with the descriptions in the literature of oxalate poisoning in sheep, the analyzed cases lack direct behavioral observation and therefore observational data to support the absolute certainty of the diagnosis: this aspect represents a limitation of this work. Further studies including information on the field monitoring of animals and careful analysis of pastures are needed to obtain a greater completeness of information and definitive certainty of causality.

## 6. Conclusions

In our work, the first episode analyzed involved a high number of animals simultaneously; it is considered a case of acute intoxication due to the way in which the episode unfolded, the observed lesions, and the severe hypocalcemia found in urine tests, as reported in the literature [7,15].

The analyses carried out on the dead animals and the study of the distribution of the episodes over time led the authors to consider the second episode a case of chronic intoxication, in which two deaths occurred spaced apart in time. Furthermore, several animals with symptoms attributable to *Oxalis pes-caprae* poisoning were found on the farm, such as weight loss and depression, which lasted for several months. In fact, pathological analyses of both dead sheep highlighted signs of a cachectic state; this result supports the hypothesis of chronic intoxication. Regarding this, some authors claim that malnourished ruminants are more susceptible to oxalate poisoning [27].

Since hypocalcemia is the most significant alteration in acute oxalate intoxication, parenteral injection of calcium salts is considered the specific treatment. For this purpose, subcutaneous or intravenous administration of 50–100 mL of 25% calcium borogluconate is effective and leads to rapid healing [20,27]. Supportive treatment is also recommended to prevent nephrosis caused by the precipitation of oxalate crystals, such as oral administration of dicalcium phosphate to bind soluble oxalate in the intestine [20].

In Sardinia, animals are usually led to pasture during daylight hours. In particular, in 2024, pastures were particularly rich in herbaceous species, some of which were potentially poisonous, due to the spring temperatures and low rainfall recorded for most of the year. In the cases described above, farmers reported that *Oxalis pes-caprae* was the predominant herbaceous plant in these pastures; grazing animals ingested large quantities of it due to the lack of other types of grass.

We therefore believe that in the above cases, there is poor management of breeding and little attention paid to the animals’ feeding, a consequence of the poor training of the breeders.

It is appropriate to make this type of problem known in order to try to contain it and reduce as much as possible the consequences due to the ingestion of large quantities of poisonous herbaceous plants. Furthermore, it is essential to underline the importance of good livestock and pasture management to minimize cases of acute and chronic intoxication resulting from the ingestion of poisonous plants: this is the purpose of this manuscript. When such cases occur, we suggest performing both direct behavioral investigations on the animals involved and grazing analyses simultaneously with clinical and pathological investigations, to obtain the necessary information to complete the diagnostic picture.

In conclusion, to the best of our knowledge, this work represents the first reported case of oxalate poisoning due to the ingestion of *Oxalis pes-caprae* in Sardinian sheep, which we hope will act as an incentive to promote appropriate dietary and management practices to mitigate the risks associated with this plant.

## Figures and Tables

**Table 1:** Analyzed samples and the laboratories that performed the analyses on dead animals.

	CASE 1	CASE 2	
	February 2024 (2 *)	March 2024 (1 *)	July 2024 (1 *)
Pathological Anatomy and Clinical Diagnostics Laboratory of the Cagliari Section of the IZS Sardinia	Necropsy	Necropsy	Necropsy
Pathological Anatomy and Clinical Diagnostics Laboratory of the Cagliari Section of the IZS Sardinia	Culture tests (brain and mesenteric lymph node)	Culture tests (brain, right and left kidney, intestine, lung, liver, and mesenteric lymph node)	Culture tests (brain, liver, kidney, and mesenteric lymph node)
Anatomy-Histopathology and Animal Genetics Laboratory of the Sassari Section of the IZS Sardinia	Histological analysis (kidney and liver)	Histological analysis (right and left kidney and liver)	Histological analysis (right and left kidney, liver, and tricuspid valve)
Animal Housing and Welfare Laboratory of Sassari Section of the IZS Sardinia	Biochemical parameters (urine)	Biochemical parameters (blood)	**-**

* Number of sheep delivered to IZS Sardinia for laboratory diagnostic tests and necropsy.

**Table 2:** Biochemical parameter analyses performed on urine samples.

	Analyzed Parameter	Result	Reference Range
Dead sheep 1	Urea	158	25–60 mg/dL
Dead sheep 2		150	
Dead sheep 1	Calcium	1.5	8–12 mg/dL
Dead sheep 2		1.4	

**Table 3:** Biochemical parameter analyses performed on blood samples.

Analyzed Parameter	Result	Reference Range
ALBUMIN	1.9	2–3.5 g/dL
ALP (Alkaline phosphatase)	120	45–250 u/L
TOTAL BILIRUBIN	0.4	0.15–0.65 mg/dL
CALCIUM	10	8–12 mg/dL
CREATININE	7.18 *	0.3–0.9 mg/dL
PHOSPHORUS	16.3 *	4–8 mg/dL
TOTAL PROTEIN	7	6–8.5 g/dL
GGT (Gamma-glutamyl transferase)	542 *	60–120 u/L
AST (Aspartate aminotransferase)	−3 *	70–200 u/L
ALT (Alanine aminotransferase)	77 *	15–45 u/L
UREA	464 *	25–60 mg/dL
CHOLESTEROL	91	30–80 mg/dL
CPK (Creatine phosphokinase)	2630 *	100–350 u/L
GLUCOSE	31	40–75 mg/dL
TRIGLYCERIDES	91	5–50 mg/dL

* Altered parameters.

**Table 4:** Altered parameters in live animals are marked with *.

	Analyzed Parameter	Result	Reference Range
Sheep 1	Calcium	8.3	8–12 mg/dL
	Urea	23 *	25–60 mg/dL
	PLT (platelet)	278 *	466–1085 [10^3^/µL]
Sheep 2	Calcium	7.5 *	8–12 mg/dL
	Urea	111 *	25–60 mg/dL
	PLT (platelet)	507	466–1085 [10^3^/µL]
Sheep 3	Calcium	9.1	8–12 mg/dL
	Urea	12 *	25–60 mg/dL
	PLT (platelet)	233 *	466–1085 [10^3^/µL]
Sheep 4	Calcium	8.3	8–12 mg/dL
	Urea	25	25–60 mg/dL
	PLT (platelet)	294 *	466–1085 [10^3^/µL]
Sheep 5	Calcium	8.9	8–12 mg/dL
	Urea	32	25–60 mg/dL
	PLT (platelet)	437 *	466–1085 [10^3^/µL]
Mean ± SD of parameters	Calcium	8.42 ± 0.6	8–12 mg/dL
	Urea	40.6 ± 42.3	25–60 mg/dL
	PLT (platelet)	349.8 ± 116.4	466–1085 [10^3^/µL]

## Data Availability

The original contributions presented in this study are included in the article. Further inquiries can be directed to the corresponding author.
